# Self-rated health status and illiteracy as death predictors in a Brazilian cohort

**DOI:** 10.1371/journal.pone.0200501

**Published:** 2018-07-12

**Authors:** Sayuri Inuzuka, Paulo Cesar Veiga Jardim, Shafika Abrahams-Gessel, Ludimila Garcia Souza, Ana Carolina Rezende, Naiana Borges Perillo, Samanta Garcia Souza, Ymara Cássia Luciana Araújo, Rogério Orlow Oliveira, Weimar Sebba Barroso, Andréa Cristina Sousa, Ana Luiza Lima Sousa, Thiago Veiga Jardim

**Affiliations:** 1 Hypertension League–Federal University of Goiás (UFG), Goiânia, Brazil; 2 Center for Health Decision Science, Harvard TH Chan School of Public Health—Department of Health Policy and Management, Boston, MA, United States of America; 3 Division of Cardiovascular Medicine, Brigham & Women’s Hospital, Boston, MA, United States of America; Edith Cowan University, AUSTRALIA

## Abstract

Cohort studies assessing predictive values of self-rated health (SRH) and illiteracy on mortality in low-to-middle income countries are missing in the literature. Aiming to determine if these two variables were death predictors, an observational prospective population-based cohort study was conducted in a Brazilian small city. The cohort was established in 2002 with a representative sample of adults living in the city, and re-assessed in 2015. Sociodemographic (including illiteracy), anthropometric, lifestyle, previous CVD, and SRH data were collected. Cox proportional hazard models were designed to assess SRH and illiteracy in 2002 as death (all causes, CVD and non-CVD) predictors in 2015. From a total of 1066 individuals included in this study, 95(9%) died of non-CVD causes and 53(5%) from CVD causes. Mortality rates were higher among those with worse SRH in comparison to better health status categories for all causes of death, CVD and non-CVD deaths (p<0.001 for all outcomes). Similarly, illiterate individuals had higher mortality rates in comparison to non-illiterate for all causes of death (p<0.001), CVD (p = 0.004) and non-CVD death (p<0.001). Higher SRH negatively predicted CVD death (HR 0.44; 95%CI 0.44–0.95; p = 0.027) and all causes of death (OR 0.40; 95%CI 0.20–0.78; p = 0.008) while illiteracy positively predicted Non-CVD death (OR 1.59; 95%CI 1.03–2.54; p = 0.046). In conclusion, we found in this large Brazilian cohort followed for 13 years that better health perception was a negative predictor of death from all causes and CVD deaths, while illiteracy was a positive predictor of non-CVD deaths.

## Introduction

Most middle income countries, including Brazil, have experienced continuous life expectancy gains in the past 30 years, following the progression of demographic and epidemiological transitions[1, 2]. Nevertheless, properly identifying individuals with reduced life expectancy is a public health priority and a central issue in clinical decision making[3] in these limited resources settings. Despite that, most epidemiological studies assessing the impact of different risk factors on mortality have mainly been conducted in high-income countries[4–8].

Self-rated health status (SRH) is a subjective measure that is being used to monitor various aspects of populations’ health[9]. Although some aspects of SRH are not fully understood, it reflects an individual’s comprehensive perception of health, which includes biological, psychological and social aspects that cannot be totally captured by outside observers[10] [11]. Recent advances in treatment strategies and secondary prevention measures driven by SRH improved cardiovascular disease (CVD) patient’s survival[12]. SRH is important not only in patients with heart disease, but is also an independent risk factor for mortality from all causes and other health outcomes[13].

Illiteracy—the lack of any or low levels of education—is also associated with higher mortality from all-causes and CVD causes[14]. Low levels of education are associated with poor or even incorrect knowledge about effective measures to prevent diseases[14], while good health is directly associated with higher levels of education[15]. Reductions in adult mortality have been associated with higher levels of education[16].

SRH and illiteracy as predictors of mortality are strongly moderated by demographic (e.g. age and sex) and socioeconomic variables (e.g. income)[17–19]. Cohort studies addressing the predictive value of these two risk factors on mortality have been conducted in developed countries and, additionally, the combined effect of SRH and illiteracy has not yet been assessed. Therefore, evidence for the predictive value of SRH and illiteracy on mortality in low-to-middle income countries are needed.

The objective of this study was to determine if self-rated health status and illiteracy were predictors of cardiovascular, non-cardiovascular, and all-causes of mortality in a longitudinal cohort of more than 1,000 individuals in a middle-income country with approximately 13 years of follow-up data.

## Materials and methods

This study is based on the second phase of data collection for an observational prospective population-based cohort study that was conducted in *Firminópolis*, a small city in the Midwest Region of Brazil. The cohort was established in 2002 (phase 1) when a representative sample of adults (≥ 18 years) living in the urban part of the town was selected[20].

Sample size in phase 1 was calculated based on: total city population in 2002 (9666 inhabitants), hypertension prevalence of 25%, 95% confidence interval and estimation error of 10%. These parameters resulted in a sample of 1030 individuals. Accounting for potential losses of follow-up, an additional 20% was added, resulting in a final sample size of 1,236[20].

Initially, a sample of urban households in the city was identified through random selection. Then individual households were selected by clusters using probabilistic sampling to ensure a representative population. Finally, in each household only one person—randomly selected from the residents who were ≥18 years old—was interviewed to avoid problems of information interdependence between interviewees. Pregnant women and mothers of children under six months of age were excluded from the lot drawing to avoid errors in data interpretation. Hospitalized residents were also excluded. The final study sample in phase 1 was 1,167 individuals (430 men and 737 women)[20, 21].

All individuals included in phase 1 with complete information on vital status at the start of phase 2 (13 years after phase 1) were included in this study, while individuals for whom no information could be obtained were excluded.

Sociodemographic, anthropometric, lifestyle variables, previous cardiovascular diseases, and SRH were assessed for individuals in Phase 1. Additionally, during phase 2, information about vital status was obtained.

Sex, age, marital status (with or without partner) and income (monthly household income in units of minimum wage) were the sociodemographic characteristics assessed. Weight, height, body mass index (BMI) and waist circumference (WC) were obtained as described in detail previously [22].

Aiming to avoid observer bias, semi-automatic devices (OMRON, HEM 705 CP) were used to measure blood pressure (BP). Measurements were performed twice: at the beginning and at the end of the interview, with a minimum interval of 5 minutes between the two measurements. The second BP measurements were used in the analyses. Hypertension was defined as systolic blood pressure (SBP) ≥ 140 mmHg and/or diastolic blood pressure (DBP) ≥ 90 mmHg, or self-reported use of anti-hypertensive medication.

Lifestyle factors measured included smoking (current smoker or non-smoker), alcohol consumption (any consumption of alcohol in last 30 days), and sedentary lifestyle (leisure, work, and commuting physical activity). When most of the reported leisure time was spent doing activities that resulted in low energy expenditure (e.g. watching TV or using a computer) individuals were considered sedentary at leisure time. Similarly, when most of the time at work was spent sitting or performing activities that involved little physical exertion individuals were considered sedentary at work. Commuting physical activity of less than 15 minutes per day was considered sedentary.

Cardiovascular diseases (CVD) were assessed by self-report and included stroke, myocardial infarction, and angina.

### Self-rated health status and illiteracy

SRH was assessed by the question: “What do you think about your current health condition?”[23]. Responses were coded using a 5-point scale (1 = poor; 2 = fair; 3 = good; 4 = very good; 5 = excellent).

Illiteracy was defined as self-report of not being able to read or write.

### Mortality outcomes

Mortality outcomes were obtained for all 1,066 individuals using: (1) Brazilian Single Health System (SUS) records, (2) urban territorial tax records of *Firminopolis’* city hall, and (3) information obtained from key members of local community, such as health care providers, school teachers, and relatives who lived near individuals in phase 1. Mortality data on all participants were confirmed at the registry office located in *Firminópolis* and *São Luis dos Montes Belos (*a city near *Firminópolis)*. All deaths were re-confirmed using the Mortality Information System of the Ministry of Health (SIM), which also listed the cause of death, coded using the International Classification of Diseases, Tenth Revision (ICD-10). Mortality outcomes were categorized as cardiovascular death (ischemic heart disease, cerebrovascular disease, other cardiovascular disease: I20-I25; I60-I69; I00-I15; I26-I45; I47-I59; I70-I99) and non-cardiovascular deaths (all other causes).

### Analyses

All analyses were conducted using STATA ® V14 software. Continuous variables were expressed in mean values with standard deviations and compared using non-paired Student’s t-test, since they were normally distributed. Categorical variables were expressed as absolute numbers and percentiles, and compared using Chi-Square test. Kaplan-Meier survival curves were used to compare the mortality outcomes between sub-groups of self-rated health status (poor/fair, good and very good/excellent) and literacy (illiterate or not-illiterate). Cox proportional hazard models were built to identify which variables from phase 1 were predictors of death from all causes, cardiovascular, and non-cardiovascular death. The models were adjusted for sex, age, obesity, hypertension, sedentary lifestyle, smoking, and cardiovascular event. The results are presented using hazard ratios and 95% confidence intervals. A two-sided p value of <0.05 was taken as significant in all analyses.

### Ethics approval

The study was approved by the Ethics in Research Committee of the institution which conducted the project with the registration number 396.839. The study followed the humans’ research regulations according to the National Health Council Resolution (number 466/2012). Participants who agreed to participate signed an Inform Consent Form before data was collected. Illiterate participants indicated consent with a thumb print instead of a written signature (procedure also approved by the ethics committee).

## Results

From the original sample of 1,167 subjects who were included in the first phase, 101 were excluded from this study since no information about them could be obtained. The final sample size was 1,066 subjects.

Mean age in phase 1 was 42.7±13.8 years while in in phase 2 it increased to 56.1±13.8 years. The mean follow-up time was 13.2 years and women made up 66.3% (n = 454) of the sample. Of the 1066 individuals included in this analysis, 918 (86%) were alive, 95 (9%) died of non-CVD causes and 53 (5%) died from CVD causes.

Baseline characteristics were compared between individuals who were alive versus dead in 2015. Deceased individuals were more likely to be male, older, and had lower income at baseline compared to those still alive in 2015. Deceased individuals were also more likely to have higher WC, higher BP, higher frequency of sedentary lifestyle, hypertension, and cardiovascular events in 2002, when compared to those alive in 2015. (Table 1)

**Table 1:** Baseline cohort characteristics according to vital status in 2015 (n = 1066).

Factor	Alive	Dead	p-value
N	918	148	
Male, n (%)	318 (34.6%)	76 (51.4%)	<0.001
Age (years), mean (±SD)	41.3 (13.9)	57.32 (13.2)	<0.001
Living with a partner, n (%)	654 (71.3%)	96 (64.9%)	0.110
Monthly household income in mw^1^, mean(±SD)	2.77 (3.2)	2.18 (2.4)	0.030
Height (m), mean (±SD)	1.60 (0.1)	1.59 (0.1)	0.320
Weight (kg), mean (±SD)	65.2 (14.0)	64.9 (12.8)	0.850
Body mass index (kg/m2), mean (±SD)	25.4 (4.9)	25.7 (4.8)	0.590
Waist circumference (cm), mean (±SD)	85.2 (11.8)	89.5 (12.5)	<0.001
Systolic blood pressure (mmHg), mean (±SD)	122.2 (31.5)	137.5 (26.8)	<0.001
Diastolic blood pressure (mmHg), mean (±SD)	79.5 (25.0)	86.2 (13.5)	0.002
Overweight/obese, n (%)	449 (48.9%)	74 (50.0%)	0.810
Smoker, n (%)	206 (22.5%)	43 (29.1%)	0.079
Sedentary lifestyle, n (%)	323 (35.2%)	69 (46.6%)	0.007
Alcohol consumption, n (%)	319 (34.8%)	43 (29.1%)	0.170
Hypertension, n (%)	355 (38.7%)	101 (68.2%)	<0.001
Cardiovascular events, n (%)	16 (1.7%)	14 (9.5%)	<0.001
Acute myocardial infarction, n (%)	4 (0.4%)	7 (4.7%)	<0.001
Stroke, n (%)	6 (0.7%)	5 (3.4%)	0.002
Angina, n (%)	7 (0.8%)	2 (1.4%)	0.470

1. MW–minimum wage

Similarly, baseline characteristics of individuals stratified by cause of death (CVD versus Non-CVD) in 2015 were compared. No statistically significant differences were found in these comparisons (Table 2).

**Table 2:** Baseline cohort characteristics according to cause of death in 2015 (n = 148).

Factor	Non-CVD death	CVD death	p-value
n	95	53	
Male, n (%)	46 (48%)	30 (57%)	0.340
Age (years), mean (±SD)	57.2 (13.5)	57.58 (12.6)	0.850
Living with a partner, n (%)	57 (60%)	39 (74%)	0.097
Monthly household income in mw^1^, mean(±SD)	1.98 (1.5)	2.52 (3.6)	0.200
Height (m), mean (±SD)	1.59 (0.1)	1.59 (0.1)	0.980
Weight (kg), mean (±SD)	65.0 (13.1)	64.9 (12.3)	0.970
Body mass index (kg/m2), mean (±SD)	25.6 (4.8)	25.7 (4.8)	0.980
Waist circumference (cm), mean (±SD)	89.6 (12.6)	89.2 (12.4)	0.850
Systolic blood pressure (mmHg), mean(±SD)	135.1 (23.6)	141.8 (31.4)	0.150
Diastolic blood pressure (mmHg), mean(±SD)	86.0 (13.3)	86.5 (14.0)	0.820
Overweight/obese, n (%)	48 (51%)	26 (49%)	0.860
Smoker, n (%)	26 (27%)	17 (32%)	0.550
Sedentary lifestyle, n (%)	48 (51%)	21 (40%)	0.200
Alcohol consumption, n (%)	27 (28%)	16 (30%)	0.820
Hypertension, n (%)	65 (68%)	36 (68%)	0.950
Cardiovascular event, n (%)	7 (7%)	7 (13%)	0.240
Acute myocardial infarction, n (%)	4 (4%)	3 (6%)	0.690
Stroke, n (%)	3 (3%)	2 (4%)	0.840
Angina, n (%)	0 (0%)	2 (4%)	0.057

1. MW–minimum wage

**Fig 1:**
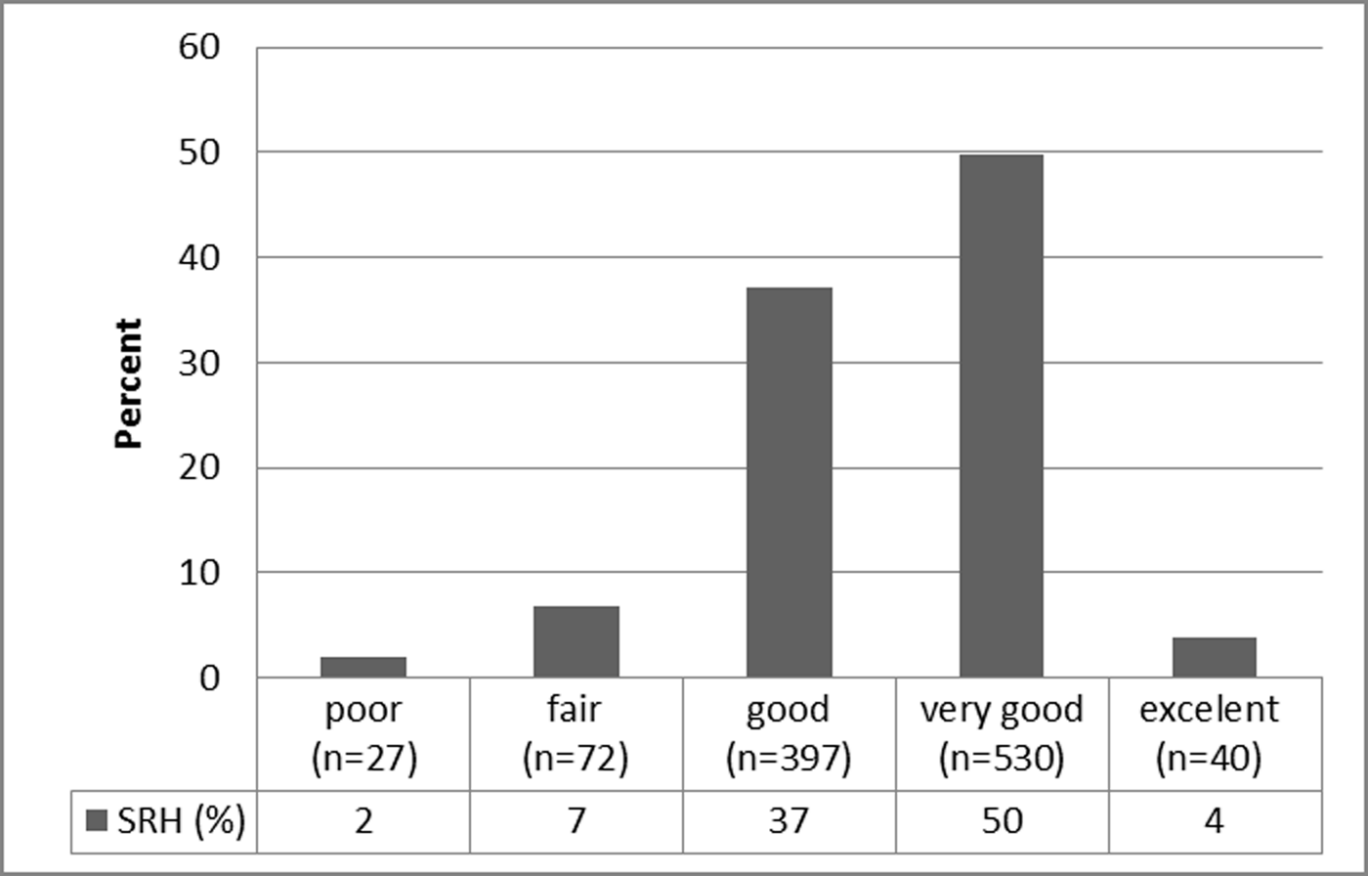
Baseline cohort self-rated health status distribution (n = 1,066).

Fig 1 shows baseline SRH distribution in the overall cohort. The majority of the population rated their health as very good (50%; n = 530) or good (37%; n = 397), while only 2% (n = 27) considered their health poor. Illiteracy was found in 14% (n = 153) of the study population.

**Fig 2:**
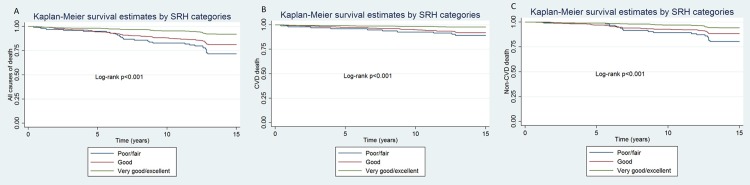
Kaplan-Meier survival curves comparing all causes of death (A), CVD death (B) and Non-CVD death (C) between sub-groups of self-rated health status (poor/fair, good and very good/excellent).

Additionally, mortality outcomes (cardiovascular, non-cardiovascular and all causes of death) were compared between sub-groups of self-rated health status. Among those with worse SRH (fair/poor) 28.3% (28 events in 99 individuals) were dead 13 years after, in comparison to 18.7% (74 events in 396 individuals) of those with good SRH and 8.1% (46 events in 570 individuals) with very good/excellent health status. Likewise, higher mortality rates among those with worse SRH in comparison to better health status categories were found for cardiovascular e non-cardiovascular deaths. Fig 2 shows the Kaplan-Meier survival curves comparing the mortality outcomes between sub-groups of self-rated health status (poor/fair, good and very good/excellent).

**Fig 3:**
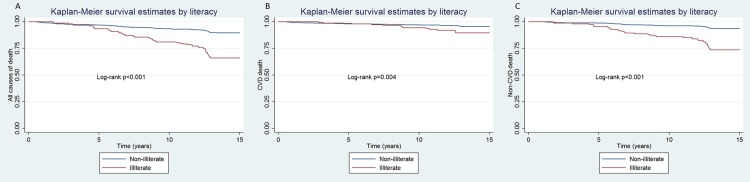
Kaplan-Meier survival curves comparing all causes of death (A), CVD death (B) and Non-CVD death (C) between illiterate and non-illiterate individuals.

Mortality comparison by illiteracy status showed that 40% (52 events in 153 individuals) of those who were illiterate were dead in 2015 in comparison to 10.5% (96 events in 913 individuals) of those not illiterate. Similar results were found for cardiovascular e non-cardiovascular death when assessed separately. The survival curves of these comparisons are shown on Fig 3.

Cox proportional hazard models built to identify if SRH and illiteracy were predictors of death from all causes, CVD and Non-CVD death in this cohort are shown in Table 3. In the adjusted models, better SRH negatively predicted CVD death and all causes of death, but had no predictive value on Non-CVD death after 13 years of follow-up. In contrast, illiteracy positively predicted Non-CVD death but had no predictive value on CVD death or on death from all causes.

**Table 3:** Simple and adjusted cox proportional hazard models assessing self-rated health status and illiteracy as predictors of death from all causes, non-CVD deaths and CVD deaths.

**Variable**	**All causes death**			
	**Simple**		**Adjusted**^1^	
	**HR (95% CI)**	***p-value***	**HR (95% CI)**	***p-value***
**SRH (better)**^2^	0.39 (0.28–0.56)	<0.001	0.65 (0.44–0.95)	0.027
**Illiteracy**	3.23 (2.31–4.53)	<0.001	1.16 (0.79–1.70)	0.459
	**Non-CVD death**			
	**Simple**		**Adjusted**^**1**^	
	**HR (95% CI)**	***p-value***	**HR (95% CI)**	***p-value***
**SRH (better)**^**2**^	0.46 (0.30–0.71)	<0.001	0.84 (0.53–1.35)	0.479
**Illiteracy**	3.98 (2.64–6.00)	<0.001	1.59 (1.03–2.54)	0.046
	**CVD death**			
	**Simple**		**Adjusted**^**1**^	
	**HR (95% CI)**	***p-value***	**HR (95% CI)**	***p-value***
**SRH (better)**^**2**^	0.28 (0.15–0.53)	<0.001	0.40 (0.20–0.78)	0.008
**Illiteracy**	2.14 (1.16–3.94)	0.014	1.64 (0.32–2.27)	0.204

1. Adjusted for sex, age, obesity, hypertension, sedentary lifestyle, smoking, and cardiovascular event.

2. SRH (better)–self-rated health status very good and excellent

## Discussion

In this large cohort study with 13 years of follow-up data and conducted in a small Brazilian town, we found that better health perception negatively predicted death from all causes and CVD deaths, while illiteracy was a predictor of non-CVD deaths. To our knowledge, this is the first longitudinal cohort study addressing SRH and illiteracy in combination as determinants of mortality.

Predictors of overweight/obesity[22] and hypertension[21] were recently assessed using data from this same cohort. Differing from our study, neither one of these studies included mortality data as the main outcome. The combination of the new knowledge provided by these publications on predictors of health related outcomes and predictors of mortality will help the healthcare community to understand the natural history of CVD and non-CVD in this low resource setting.

Our cohort had SRH and literacy characteristics similar to what has been observed in the greater Brazilian population. We observed illiteracy rates similar to the overall Brazilian rates, which ranged from 6.3% to 33.4% depending on the country region assessed[24]. There were fewer individuals reporting fair and poor SRH, and more reporting very good and excellent SRH at baseline in our study when compared to cross-sectional studies conducted in other segments of the Brazilian population at the approximately same time period[25, 26]. This is likely explained by the fact that our study was conducted in a small country town with better living conditions, particularly in comparison to large urban centers from developing countries.

Studies conducted in different countries with several follow-up periods showed that individuals with poor health assessed by SRH had a higher mortality risk than those with excellent health, even after adjustments for chronic diseases [9, 25, 27, 28]. The association between SRH and death is more plausible for chronic conditions, with a long-term course and greater impact in daily life, when compared to more aggressive diseases with worse prognosis in the medium-term[29]. We found that those who report themselves with very good or excellent health had less chance of dying from CVD and all causes.

SRH is widely used as a measurement of health given it consistency as a mortality predictor but mostly its simplicity and applicability. Besides that, differences on SRH accuracy have been reported in the literature, with more reliable results found in white individuals, higher income groups and populations with higher levels of education, for example[30–32]. These differences need to be taken into consideration when interpreting our results, particularly considering that our study was conducted in a population with low-income, high prevalence of illiteracy and in country where the population is mostly non-white. Notwithstanding, we found higher mortality rates among those with worse SRH in comparison to better health status categories for all mortality outcomes, which is consistent with the literature [9, 25, 27, 28].

Cohort studies demonstrated that those with higher education level have greater age-specific survival probabilities compared to those with lower education levels[33] and suggested that mortality could be reduced if the levels of CVD risk factors were reduced in less educated people[34]. These studies were conducted in developed countries, where there are lower rates of illiteracy, better adherence to lifestyle changes, early diagnosis of chronic diseases, and lower prevalence of cardiovascular risk factors, which justify the lower CVD mortality in these countries[34–36]. Cross-sectional studies conducted in developing countries demonstrated an association between illiteracy and mortality from all-causes, with a tendency to increase CVD mortality[37, 38]. Illiteracy predicted Non-CVD death in our study, but not CVD deaths and deaths from all causes.

A potential limitation of this study is the fact that 101 individuals from phase 1 were not found during the second phase of the study and were excluded. Although this follow-up loss represents less than 1% a year, information bias due to a lack of outcome data for these individuals could have affected the final analysis. We addressed this limitation by comparing the baseline characteristics (sex, age, marital status, income, overweight/obesity, smoking, sedentary lifestyle, alcohol consumption, hypertension, and cardiovascular events) of individuals not found with those with completed follow-up. No statistically significant differences were observed between the groups’ characteristics (S1 Table).

Another limitation is the assessment of illiteracy exclusively by self-reports. Using standardized measurement methods, such as Rapid Estimate of Adult Literacy in Medicine (REALM)[39] or even some measure of formal education could be more informative. Nevertheless, the use of self-reported illiteracy in large epidemiological studies has been reported in the literature with consistent results [40, 41].

Most data relating SRH and illiteracy with mortality come from developed countries. Studies conducted in developing countries either used cross-sectional data to address this relation or assessed these variables separately[42, 43]. We conducted a longitudinal study in a developing country and, additionally, assessed SRH and illiteracy jointly in the regression models.

Although some primary care interventions to reduce mortality showed improvements in SRH after 10 years[44], their applicability in developing countries have yet to be determined. Nevertheless, SRH could be used to determine which population groups should have easy access to health services and monitoring programs to reduce CV deaths. Additionally, educational programs should be developed to eradicate illiteracy and to improve the population’s health knowledge as a way to reduce non-CVD morbidity and mortality, and consequently reduce health related costs. The association between illiteracy and non-CVD death may be reduced with primary care improvements, such as education, sanitation and healthcare access.

## Conclusion

In conclusion, better self-rated health status was a negative predictor of CVD death and all causes of death but had no predictive value for non-CVD death, while illiteracy predicted Non-CVD death but had no predictive value for CVD death or death from all causes after 13 years of follow-up in a Brazilian cohort.

## Supporting information

S1 Table Baseline cohort characteristics comparison between individuals with complete follow-up and those not found in 2015 (n = 1167). (DOCX)

S1 File Renamed_660b8.xls. (XLS)
