# Supplementary material for: Self-rated health status and illiteracy as death predictors in a Brazilian cohort
Source: PLoS One. 2018 Jul 12;13(7):e0200501. doi: 10.1371/journal.pone.0200501 (PMC6042772; doi:10.1371/journal.pone.0200501)
Supplement: S1 Table — (DOCX) [file pone.0200501.s001.docx]

**S1 Table. Baseline cohort characteristics comparison between individuals with complete follow-up and those not found in 2015 (n=1167).**

| **Factor** | **Complete follow-up** | **Not found** | **p-value** |
| --- | --- | --- | --- |
| **N** | 1066 | 101 |  |
| **Male, n (%)** | 394 (37.0%) | 39 (38.1%) | 0.650 |
| **Age (years), mean (±SD)** | 43.56 (14.9) | 41.1 (15.0) | 0.610 |
| **Living with a partner, n (%)** | 750 (70.4%) | 68 (67.3%) | 0.310 |
| **Monthly household income in mw^1^, mean(±SD)** | 2.68 (3.1) | 2.5 (3.1) | 0.940 |
| **Overweight/obese, n (%)** | 523 (49.1%) | 50 (49.5%) | 0.939 |
| **Smoker, n (%)** | 249 (23.4%) | 23 (22.8%) | 0.892 |
| **Sedentary lifestyle, n (%)** | 392 (36.8%) | 41 (40.6%) | 0.450 |
| **Alcohol consumption, n (%)** | 362 (34.0%) | 33 (32.7%) | 0.792 |
| **Hypertension, n (%)** | 456 (42.8%) | 47 (42.6%) | 0.969 |
| **Cardiovascular events, n (%)** | 30 (2.8%) | 3 (3.0%) | 0.574 |
| **Acute myocardial infarction, n (%)** | 11 (1.0%) | 1 (1.0%) | 0.270 |
| **Stroke, n (%)** | 11 (1.0%) | 1 (1.0%) | 0.190 |
| **Angina, n (%)** | 9 (0.8%) | 1 (1.0%) | 0.330 |

1. MW – minimum wage
